# ARF19 Condensation in the Arabidopsis Stomatal Lineage

**DOI:** 10.17912/micropub.biology.000708

**Published:** 2023-02-04

**Authors:** Chi Kuan, Lucia C. Strader, Nicholas Morffy

**Affiliations:** 1 Department of Biology, Duke University, Durham, North Carolina 27708, USA

## Abstract

The phytohormone auxin regulates nearly every aspect of plant development. Transcriptional responses to auxin are driven by the activities of the AUXIN RESPONSE FACTOR family of transcription factors. ARF19 (AT1G19220) is critical in the auxin signaling pathway and has previously been shown to undergo protein condensation to tune auxin responses in the root. However, ARF19 condensation dynamics in other organs has not yet been described. In the Arabidopsis stomatal lineage, we found that ARF19 cytoplasmic condensates are enriched in guard cells and pavement cells, terminally differentiated cells in the leaf epidermis. This result is consistent with previous studies showing ARF19 condensation in mature root tissues. Our data reveal that the sequestration of ARF19 into cytoplasmic condensation in differentiated leaf epidermal cells is similar to root-specific condensation patterns.

**Figure 1. ARF19 condensation differs in distinct cell types in the Arabidopsis cotyledon epidermis.**
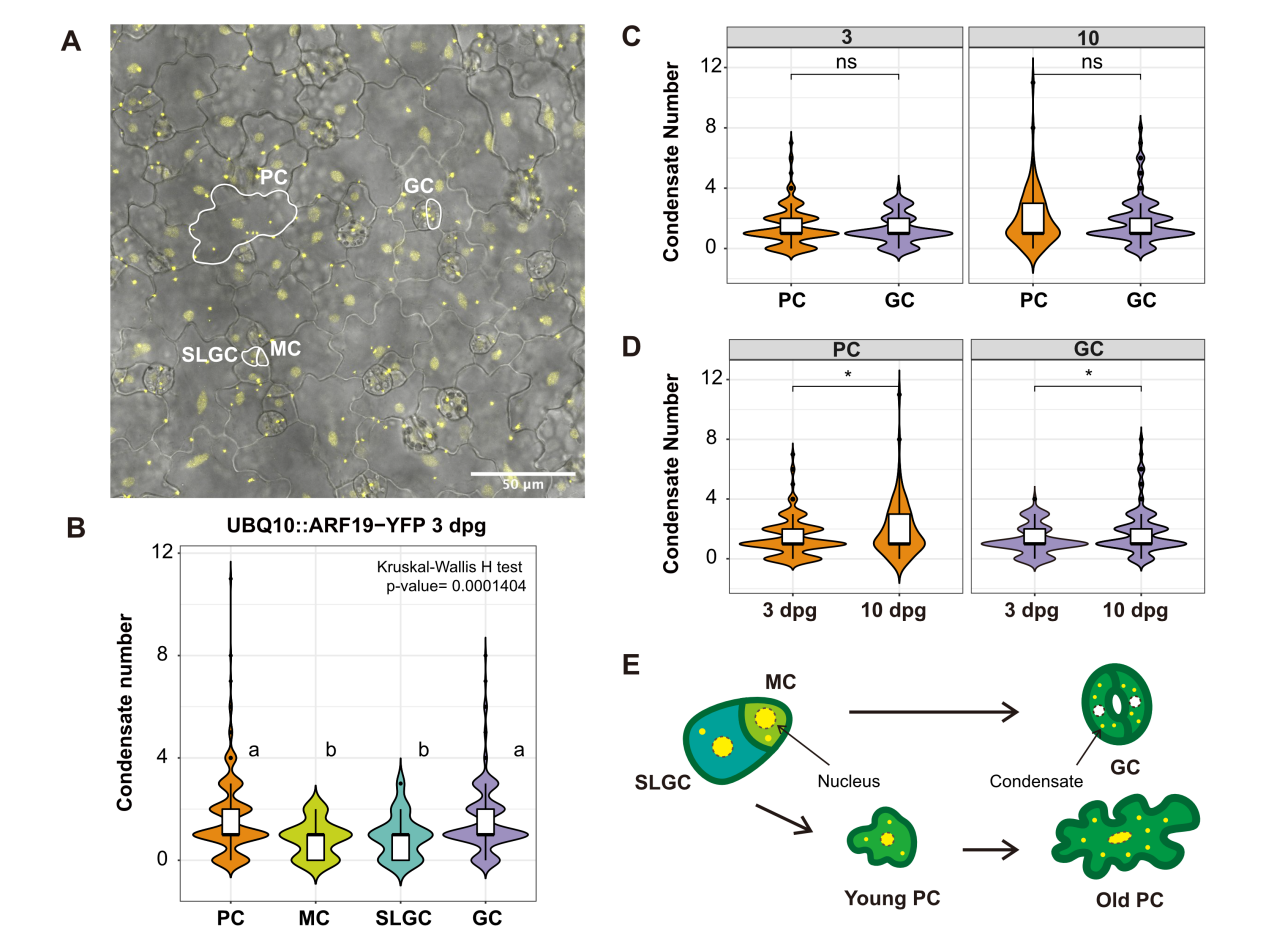
**(A)** Representative confocal image of ARF19-YFP in the cotyledon epidermis of Arabidopsis seedlings 3 days post germination (dpg). The four measured cell types, pavement cell (PC), meristemoid cell (MC), stomatal lineage ground cell (SLGC) and guard cell (GC), are outlined in white. The image was merged from the bright field channel and the maximum intensity projection of z stacks in the YFP channel. **(B)** The graph depicts ARF19 condensate numbers in different cotyledon epidermal cell types 3dpg. Letters represent statistical groupings based on pairwise comparisons using Wilcoxon rank sum test with continuity correction with a p-value of 0.05. At least 200 cells from 3 individuals were assayed. **(C)** and **(D)** show violin plots comparing condensate number in PC and GC at 3 and 10 dpg. The * indicates significant difference at *P* < 0.05 between cell types at 3 and 10 dpg **(C)** and within cell types at 3 and 10 dpg **(D)** by Wilcoxon rank sum test. At least 80 cells of each cell types from 3 individuals were counted. **(E)** Model of ARF19 condensation in the cotyledon epidermis. In dividing, undifferentiated cell types ARF19 is found primarily in the nucleus (outlined cellular compartment) and not in condensates (yellow spots). Following differentiation into GC and PC, ARF19 condensate abundance increases and is depleted in the nucleus.

## Description

How plants control developmental processes is an enduring question for plant biologists. For the past century, researchers have studied how phytohormones and other signaling molecules affect developmental responses. One of these phytohormones, auxin, plays a crucial role in plant morphogenesis and growth by responding to intrinsic and external cues (Zhao 2010). A family of plant-specific transcription factors called AUXIN RESPONSE FACTORS (ARFs) modulate auxin signal transduction (Korasick *et al.* 2014). AUXIN RESPONSE FACTOR 19 (ARF19) is a transcriptional activator that regulates auxin-mediated transcription and developmental responses in *Arabidopsis thaliana* (Okushima *et al.* 2005). Recent work has shown that ARF19 is found in the nucleus of actively dividing cells in the root meristematic zone and localized to biomolecular condensates that reside in the cytoplasm of mature cells (Powers *et al.* 2019, Jing *et al.* 2022). Biomolecular condensates are membraneless and nonstoichiometric compartments consisting of one or more biological molecules. Because condensation is dependent on many environmental parameters and can regulate cellular processes, it plays important roles in plant development (Emenecker *et al.* 2021). In Arabidopsis roots, meristematic and dividing cells show nuclear ARF19 localization and have increased expression of the auxin signaling reporter, DR5 (reviewed in Jedličková *et al.* 2022), whereas mature cells have cytoplasmic ARF19 condensates and lack DR5 expression, suggesting attenuated auxin responses in these tissues (Powers *et al.* 2019). When ARF19 variants that prevent condensation are expressed in Arabidopsis, DR5 expression is restored in all root tissues, suggesting that ARF19 condensation contributes to the attenuation of auxin responses (Powers *et al.* 2019, reviewed in *Morffy and Strader* 2022). It is unknown whether the nucleo-cytoplasmic partitioning of ARF19 occurs only in the Arabidopsis root or if it is a regulatory mechanism in other organs.

Because the leaf epidermis is a well-studied and auxin-dependent cell development system, it is an ideal target to observe ARF19 condensation behavior. There are four primary cell types in the leaf epidermis: (1) Pavement cell (PC), a puzzle-shaped cell for forming a protective layer of the leaf. (2) Meristemoid cell (MC), a triangular stomatal precursor cell with self-renewing ability. (3) Stomatal Lineage Ground Cell (SLGC), a larger sister cell of MC with mixed cell type potential. (4) Guard cells (GC), mature cells with a pore controlling gas exchange. MC and SLGC form after asymmetric cell division (ACD) from leaf protodermal cell. MC, the small daughter cell that forms after the ACD, gains the stomatal fate and becomes the precursor of stoma. Then, stoma forms through the symmetric cell division of MC into two GCs. SLGC, the large daughter cell that forms after the ACD, is a biopotent cell that can turn into a pavement cell or undergo another round of ACD and differentiate into guard cells (Lee and Bergmann 2019). The distribution of auxin signaling in the epidermis affects the epidermal patterning, and auxin depletion is required for gaining the stomatal fate (Le *et al.* 2014).

We hypothesized that ARF19 cytoplasmic condensate number would be higher in terminally differentiated cells in the leaf epidermis, where auxin signaling is reduced (Le *et al. 2014* , Grones *et al.* 2020), consistent with their localization pattern in root tissues. We observed the cotyledons of transgenic Arabidopsis seedlings expressing YFP-tagged ARF19 under the *UBQ10* promoter three days post germination (dpg) using confocal microscopy and quantified ARF19 condensates in PC, MC, SLGC, and GC cell types (Fig 1A). The dividing cell types, MC and SLGC, had fewer ARF19 condensates than the differentiated cell types, GC and PC, consistent with ARF19 condensation patterns in the root (Fig 1B) (Powers *et al.* 2019).

To test whether condensate number is related to developmental stage, we compared ARF19 condensate numbers in PC and GC between 3 dpg and 10 dpg. ARF19 condensate numbers were similar in both cell types at both time points (Figure 1C). However, when we compared condensate numbers within the same cell type between timepoints, we found that the number of condensates increased at 10 dpg (Figure 1D). These results suggest that differentiated cells accumulate ARF19 condensates as they mature.

Together, these data provide the evidence for a leaf model where ARF19 is in the nucleus of dividing cells such as MC and SLGC, but are sequestered in cytoplasmic condensates in differentiated cells, like PC and GC (Fig. 1E). This pattern of ARF19 cytoplasmic condensation in PC and GC correlates with the absence of DR5 expression in these cell types (Le *et al.* 2014, Grones *et al.* 2020), suggesting ARF19 cytoplasmic condensation and nucleo-cytoplasmic partitioning in the cotyledon epidermis results in attenuated auxin signaling, consistent with its localization patterns in root tissues. The distinct regulation of ARF19 condensation may provide a mechanism to tune auxin responses during leaf epidermis development, in addition to other mechanisms such as PIN localization and auxin efflux (Le *et al.* 2014, Grones *et al.* 2020). However, we cannot rule out additional factors that may affect ARF19 condensation in the leaf epidermis. First, because we used the *UBQ10* promoter, ARF19 transcriptional dynamics may not be identical to the native expression patterns. Although Powers *et al.* (2019) suggested the tissue-specific differences of ARF19 condensation in root were not related to gene expression levels in the root, it remains to be seen if this is also true in the leaf epidermis. Second, the experiments were conducted using cotyledon epidermis, an embryonically developed organ. True leaves that develop post embryonically may have different ARF19 condensation and nucleo-ctyoplasmic partitioning patterns. Thus, further study of ARF19 condensation in true leaves is necessary to determine the universality of condensation-mediated attenuation of auxin response.

## Methods

**Plant lines and growth conditions**

ARF19 condensates were quantified from *Arabidopsis thaliana* Col-0 carrying UBQ10p::YFP- ARF19 (Power *et al.* 2019). Seeds were surface sterilized with 20% (v/v) bleach and 0.01% (v/v) Triton X-100. After rising 4 times with sterilized water, the sterilized seeds were suspended in 0.1% agar and stratified for 2 days at 4°C. Stratified seeds were plated in plant nutrient (PN) media (Haughn and Somerville 1986) with 0.6% agar and supplemented with 0.5% (W/V) sucrose at 22°C under continuous white light (GE fluorescent lamp (F17T8/SP41/ECO) at 120 µmol m ^-2^ s ^-1^ ).

**Confocal imaging**

To quantify ARF19 condensates, 3 dpg and 10 dpg cotyledons were cut and mounted in water under coverslip. All images were collected from Leica SP8 confocal microscope with HC PL APO CS2 40X/1.0 water immersion objective, 1024X1024 pixels format, and 700 Hz speed. The images were acquired by unidirectional scanning with a voxel size of 0.221μm X 0.221μm X 1 μm. YFP were excited at 514-nm and detected at 519-547 nm with HyD detector. The Z stack covers all the epidermal cells. We collected at least 40 cells for each cells types from at least 3 individual seedlings.

**Image processing and statistical analysis**

Using FIJI (Schindelin *et al.* 2012), we performed maximum intensity projection of z stacks of the YFP channel. Guard cells, meristemoids, stomatal lineage ground cells, and pavement cells were categorized based on their morphology. Guard cells are kidney-shaped cells. Meristemoids are smaller daughter cells after asymmetric division with triangular shapes. Stomatal lineage ground cells are bigger daughter cells after asymmetric division. Pavement cells are jigsaw- shaped cells. Condensate numbers were quantified based on the projection results. Kruskal-Wallis H test and pairwise comparison with Wilcoxon rank sum test for Fig. 1B was done in R 4.0.4 (R Core Team, 2021). Wilcoxon signed-rank test for Fig .1C and D and all the violin plots were generated by the ggpubr (v0.4.0, Wickham) package in R 4.0.4.
